# Male‐Biased Dispersal Leads to Expansion After Decline: The Hokkaido Brown Bear ( *Ursus arctos* ) Population Rebounds Post‐Last Glacial Period

**DOI:** 10.1002/ece3.74312

**Published:** 2026-09-20

**Authors:** Yu Endo, Naoki Osada, Tsutomu Mano, Atsushi J. Nagano, Ryuichi Masuda

**Affiliations:** ^1^ Department of Natural History Sciences, Graduate School of Science Hokkaido University Sapporo Japan; ^2^ Faculty of Environmental Science, Nagasaki University Nagasaki Japan; ^3^ Faculty of Information Science and Technology Hokkaido University Sapporo Japan; ^4^ Research Institute of Energy, Environment and Geology Hokkaido Research Organization Sapporo Japan; ^5^ Faculty of Agriculture Ryukoku University Otsu Japan; ^6^ Institute for Advanced Biosciences Keio University Yamagata Japan; ^7^ Department of Biological Sciences Faculty of Science, Hokkaido University Sapporo Japan

**Keywords:** ddRAD‐seq, landscape genetic, last glacial maximum, mitochondrial DNA, population demography, Sex‐biased dispersal

## Abstract

Sex‐biased dispersal potentially underpins the genetic structural mismatch in genetic structure between differently inherited genetic loci, but accurately evaluating the effect of sex‐biased dispersal is challenging due to other sex‐biased traits and limitations in usable data. Brown bears exhibit extreme male‐biased dispersal, with significant genetic differences between maternally and paternally inherited loci. This study aims to investigate how the characteristic genetic structure in Hokkaido, Japan has been shaped and to evaluate the influence of male‐biased dispersal and environmental barriers on this process. We conducted a reduced‐representation genome sequencing, ddRAD‐seq, and combined it with mitochondrial DNA and Y chromosomal haplotype data. Genetic differences between the southern subpopulations and the other subpopulations were observed, which may reflect restricted gene flow, particularly among female bears, as a consequence of lowland barriers. The ratio of genetic differentiation between the X‐chromosome and autosomes suggests a significant effect of male‐biased dispersal within the Hokkaido population, with slight variation in this effect among subpopulations. Species distribution modeling estimated that suitable habitat decreased during the Last Glacial Period, and demographic simulation inferred a historical population decline during this period, followed by population expansion. These results suggest that the Hokkaido population had contracted and isolated in spite of the appearance of the land bridge and has expanded after that while being limited by the lowlands. Male‐biased dispersal likely contributed to population recolonization after significant population decline and habitat reduction due to climate changes.

## Graphical abstract

Sex‐biased dispersal potentially underpins the genetic structural mismatch in genetic structure between differently inherited genetic loci. To understand how such a mismatch has formed, we conducted genetic analysis of the brown bear in Hokkaido, Japan, with significant genetic differences between maternally and paternally inherited loci. We found that the Hokkaido population had contracted during the Last Glacial Period, and has expanded rapidly after that, mainly by males, while being limited by the lowlands.

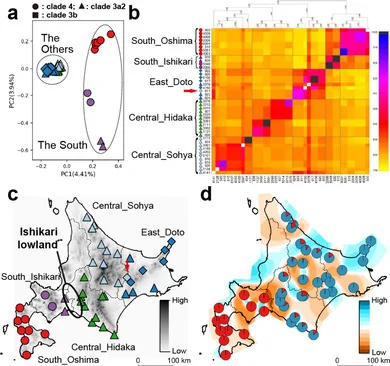

## 1 Introduction

Dispersal is a fundamental species trait that promotes the migration of novel environments and shapes genetic structures and population demography. Increased gene flow, resulting from mating in migration areas, leads to genetic homogenization between populations (de Jong et al. 2023) while reduced gene flow, caused by limited dispersal, isolates populations from each other (Frankham et al. 2010). This highlights the importance of considering the scale and rate of dispersal to understand a population's demographic history and designing effective conservation strategies for wildlife management.

The scale and rate of dispersal within species frequently differ between sexes, and this is referred to as sex‐biased dispersal (Trochet et al. 2016). In most mammals, particularly carnivores, males disperse more long distances, while females exhibit philopatry (Greenwood 1980). The scale of sex‐biased dispersal has been revealed by individual tracking (e.g., Zedrosser et al. 2007; Shirane et al. 2019), but this method is not suitable to evaluate its long‐term influence on population demographic history (Lawson Handley and Perrin 2007). Genetic analysis addresses this issue and has been proven effective in assessing this influence. Numerous studies using genetic analysis have detected the mismatch of genetic patterns between maternally inherited mitochondrial DNA (mtDNA) and the paternally inherited nonrecombining region of the Y chromosome (e.g., Andrews et al. 2013; Coia et al. 2013; Matsudaira et al. 2018; Seielstad et al. 1998). However, the process underlying this mismatch needs further investigation for several reasons. When employing a genetic approach, determining sex‐biased dispersal is complicated by various factors beyond just dispersal, including differences in society, lifespan, and sex ratio, all of which influence sex‐biased demography (Webster and Wilson Sayres 2016). Haploid genome data, which was commonly used in previous studies, made it challenging to accurately identify differences in sex‐biased dispersal because it could not pinpoint the influences of various factors, such as gene flow between groups, balancing selection on markers, and the limited resolution of markers (Bossart and Pashley Prowell 1998). Although some studies address this problem using nuclear markers (e.g., Schregel et al. 2018; Tucker et al. 2017), most evaluate sex‐biased dispersal focusing on only a single or few generations, by categorizing individuals based on sex and comparing the results between the sexes. This method is not suitable for evaluation of the long‐term difference of sex‐biased dispersal in gene flow.

The brown bear ( *Ursus arctos* ), a large member of the order Carnivora, is a model species for studying the effect of sex‐biased dispersal on population demographic history because this species exhibits extreme male‐biased dispersal, and this type of dispersal is the only confirmed sex‐biased trait that affects population demography. Males typically disperse long distances from their birthplace, while females exhibit philopatry (McLellan & Hovey, 2001; Zedrosser et al. 2007; Sato 2015). This difference is reflected in their home range size. For example, the home range sizes of adult females and males were recorded as 13.4–43.0 km^2^ and 199–496 km^2^, respectively in Hokkaido (Sato 2015). This species is solitary except during mating and rearing of offspring. In addition, there is no confirmed significant bias in the sex ratio in the population (Swenson et al. 1994) and difference in reproductive success between sexes (Shimozuru et al. 2020).

**FIGURE 1:**
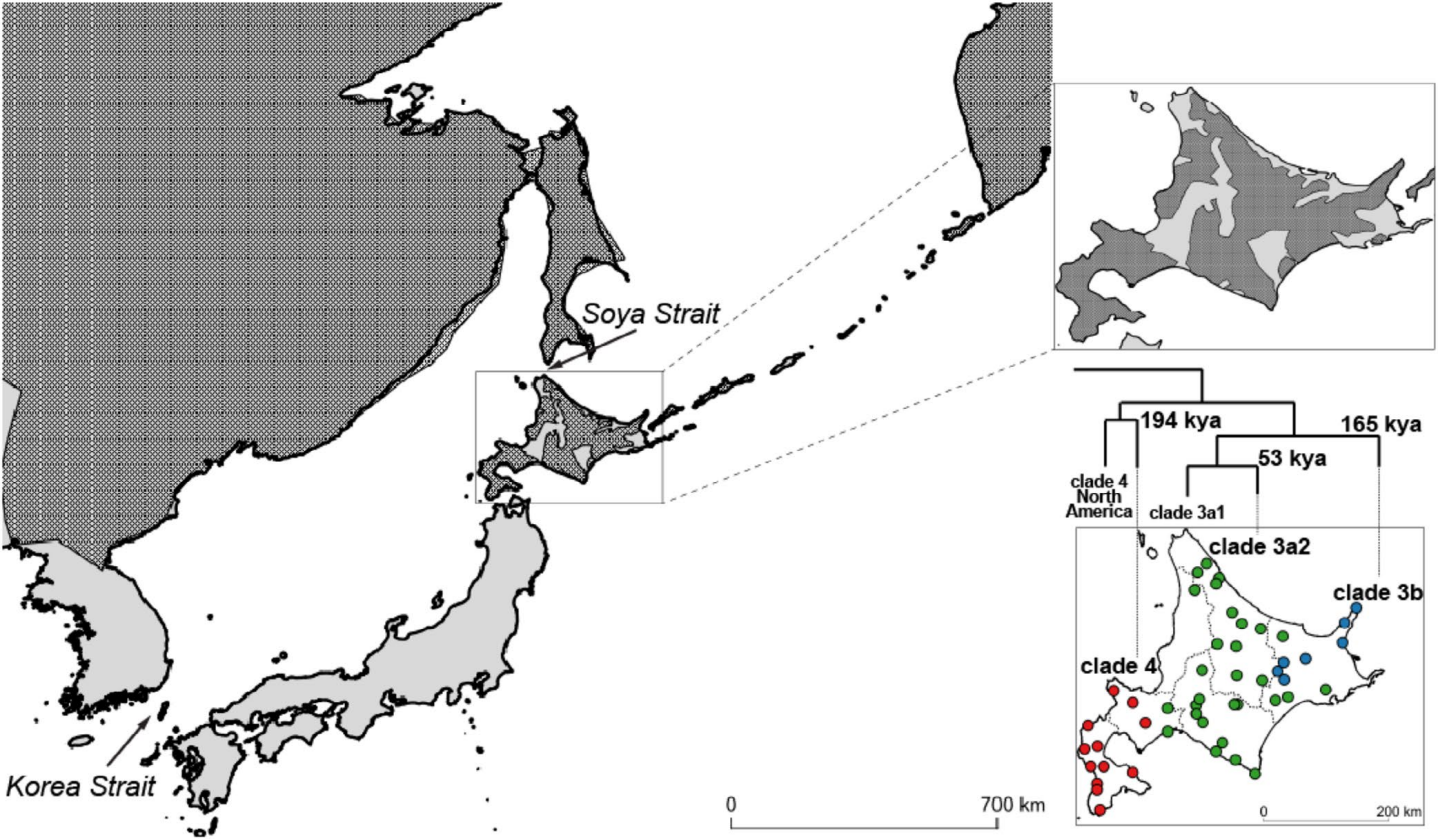
Habitat of brown bears ( *Ursus arctos* ) and sampling locations of specimens used for this study. The gray shading in the left map and the top map the distribution of brown bears (McLellan et al. 2017). The dot in the bottom‐right map colored by mt DNA lineages (red, clade 4; green, clade 3a2; blue, clade 3b) shows the sampling location of 49 individuals in this study. The dashed lines on the right map mean the management unit described by the Brown Bear Management Plan on Hokkaido (Hokkaido. 2017, 2022). The coalescence times of each lineage were estimated by Hirata et al. (2013). kya, thousands of years ago. The blanch length is not reflected genetic differentiation.

Previous studies have shown a mismatch in genetic structure between mtDNA and the Y‐chromosomal DNA, suggesting that this discrepancy is caused by incomplete lineage sorting and male‐biased dispersal (Bidon et al. 2014; de Jong et al. 2023). This mismatch has been particularly evident in Hokkaido. Matsuhashi et al. (1999) and Hirata et al. (2013) identified three deeply divergent mtDNA lineages with varying split times (clade 4: 194 kya, clade 3a2: 53 kya, clade 3b: 165 kya), each distributed across separate areas (Figure 1). In contrast, Hirata et al. (2017) found no clear genetic differentiation in Y‐chromosomal DNA haplotypes in the Hokkaido population. Thus, an apparent mismatch in genetic structures between mtDNA and Y‐chromosomal DNA within the Hokkaido population may be attributed mainly to male‐biased dispersal (Endo et al. 2021).

Genomics analysis using few whole‐genome resequencing data showed monophyly on the nuclear genome within the Hokkaido population by comparison with the continental population (Endo et al. 2021; de Jong et al. 2023), suggesting that male‐biased dispersal on migration from the Eurasian continent has led to genetic affinity within the Hokkaido population. However, the process underlying this mismatch after migration is still unknown. de Jong et al. (2023) suggested that the distinct distributions of mtDNA lineages resulted from the random fixation driven by environmental factors, such as landscape (Cushman et al. 2006) and global climate change (Hewitt 2000), because the boundaries between the mtDNA lineages coincide with the Hokkaido lowlands. To obtain the genomic evidence and detect the specific environmental factors that support the suggestion, further genome‐wide datasets for many individuals in Hokkaido and landscape analysis using the dataset are required.

In this study, we investigated the demographic history of the Hokkaido population after colonization of the island, focusing on the mismatch in genetic structure between mtDNA and Y‐chromosomal DNA that was formed. First, using double digest restriction‐site associated DNA sequencing (ddRAD‐seq) data, we analyzed the genetic structure and population demography of the Hokkaido population, focusing on autosomal and X‐chromosomal genotype data compared with previous mtDNA and Y‐chromosomal DNA data (Matsuhashi et al. 1999; Hirata et al. 2017). We then examined environmental factors, such as elevation and climate changes, that can resist dispersal and could influence migration patterns over several centuries to ten millennia. Based on these results, we assess the effect of sex‐biased dispersal on population demography by investigating the process underlying the separated distribution of three distinct mtDNA lineage clusters.

## 2 Materials and Methods

### 2.1 Brown Bears in Hokkaido and Samples

Forty‐nine brown bears from Hokkaido, collected by the Environmental and Geological Research Department, Hokkaido Research Organization, were used for the experiment (Figure 1; Table S1). Hokkaido is an island in East Asia covering approximately 83,424 km^2^ and is characterized by a subarctic climate. We selected samples from all subpopulations described as management units that correlate with subprefecture in Hokkaido (Oshima Peninsula, Shakotan‐Eniwa, Hidaka‐Yubari, Doto‐Sohya *a*, Doto‐Sohya *b*, and Teshio‐Mashike) based on the brown bear management plan of Hokkaido (Hokkaido 2017; Hokkaido 2022). All English names used for these subpopulations follow those in Sato et al. (2005). The mtDNA and Y‐chromosomal haplotypes used in this study were the same as those used in previous studies (Matsuhashi et al. 1999; Hirata et al. 2017). Current number of individuals was estimated 11,700 in 2020 (95% CI: 6600–19,300; Hokkaido 2022) and 12,200 in 2022 (95% CI: 6300–21,300; Hokkaido 2024, Sato 2026).

### 2.2 Sequencing of the Hokkaido Population

Total genomic DNA was extracted from the muscle or liver with a DNeasy Tissue & Blood Kit (QIAGEN, Hilden, Germany) or QIAamp DNA Micro Kit (QIAGEN, Hilden, Germany). The DNA concentration was measured using a Thermo Scientific NanoDrop 2000 (Thermo Scientific, Waltham, MA, USA). TE buffer (pH 8.0) was added to the DNA extractions as needed to attain a DNA concentration of 10 ng/μl.

Subsequently, we applied ddRAD‐seq to the samples. *Eco*RI and *Bgl*II were used as restriction enzymes to cut off DNA sequences following the methodology of Sakaguchi et al. (2015). The libraries were run on an Illumina HiSeq X sequencer using 150‐bp paired‐end mode at Macrogen (Kyoto, Japan). The sequence data are available from the public database (DDBJ/EMBL/NCBI accession numbers: PRJDB17752).

### 2.3 Genotyping and Site Filtering

Pair‐end sequencing data were filtered to exclude low‐quality sequences using process_radtags (option: ‐c ‐q –index_index –retain_header –disable_rad_check) in Stacks ver. 2.5 (Catchen et al. 2013). Subsequently, the data were mapped to the reference sequence of the brown bear (UrsArc2.0) using Bowtie2 ver. 2.4.4 (Langmead and Salzberg 2012) and duplicate sequences were removed using Samtools ver. 1.7 (Li et al. 2009). Genotypes were called using BCFtools ver. 1.19 (Danecek et al. 2021) mpileup with the minimum base quality filter applied using ‐Q 20 option and bcftools ver. 1.19 call including non‐variant sites.

We applied variant filtration following several steps to obtain reliable genotyping data and made four datasets (Dataset 1–4; Figure S1). First, we calculated a (30,2)‐mappability for each site, which means the 30‐mer starting at each site occurs only once in the sequence with up to 2 errors, using GenMap ver. 1.3.0 (Pockrandt et al. 2020). Sites whose mappability value is 1 and a depth is more than 3 were selected using VCFtools ver. 0.1.17 (Danecek et al. 2011).

After extracting the genotyped sites' data on unplaced scaffolds classified as autosomes in this study (scaffold_1–34, 36, 37) and X chromosome scaffolds (hap2X_scaffold_3), and selecting sites with a missing rate of less than 10% among all individuals whose missing rate was less than 20%, four datasets were created using PLINK ver. 1.90 (Purcell et al. 2007). This selected site dataset was named Dataset 1 (Figure S1). Some basic population genetic statistics, such as nucleotide diversity, were calculated using populations in Stacks. Of the four datasets, we selected sites located in non‐coding regions without missing sites for the demographic simulation using fastsimcoal2 ver. 2.6 (Figure S1, Dataset 2). All missing sites were excluded from the landscape genetic analysis (Mantel test, Partial Mantel test, and ResistanceGA; Figure S1, Dataset 3). For genetic structure analyses (Principal Component Analysis (PCA), fineRADstructure, and ADMIXTURE), sites under linkage disequilibrium (LD) and observed minor alleles less than 2 in all individuals were excluded by using the PLINK options “–indep‐pairwise 50 5 0.5.” and “–mac 2”, respectively (Figure S1, Dataset 4).

### 2.4 Genetic Structure

To investigate the genetic structure, a PCA and fineRADstructure were applied to Dataset 4 using the –pca option in PLINK and default parameters in fineRADstructure (Malinsky et al. 2018), respectively. Clustering analysis from *K* = 1 to *K* = 10 was also performed using ADMIXTURE ver. 1.3 (Alexander et al. 2009). *F*_st_ was calculated using smartpca in EIGENSTRAT (Patterson et al. 2006) without optional parameters to detect genetic differentiation between subpopulations. Pairwise *d*_xy_ was calculated in 50,000 bp windows (—window_size 50,000) using pixy ver. 2.2.0 (Korunes and Samuk 2021). The window‐based results were then averaged for each pair of subpopulations.

### 2.5 Gene Flow

We conducted two analyses, Estimated Effective Migration Surfaces (EEMS) and *Q* statistics, to evaluate gene flow between the subpopulations and between the continental populations. First, visualization of gene flow between the Hokkaido subpopulations was done using Dataset 1 and EEMS (Petkova et al. 2016) with the number of modeled demes set to be 400, and Markov chain Monte Carlo (MCMC) chains comprising 100 million iterations each were run, discarding the first 10 million generations as burn‐in and sampling every 10,000th generation. The matrix of pairwise genetic differences based on identity‐by‐state was calculated using the –1‐ibs option in PLINK. Second, *Q* statistics can be used to evaluate dispersal bias between males and females, which can then be used to evaluate the autosome‐to‐X genetic drift ratio (Chen et al. 2018; Keinan et al. 2009). If there is no bias in the effective population size or dispersal between males and females, the *Q* statistic value converges at 0.75. We used the formula *Q* = ln(1–2*F*_st_^A^)/ln(1–2*F*_st_^X^), which *F*_st_^A^ means autosomal *F*_st_ and *F*_st_^X^ means X‐chromosomal *F*_st_, following the methodologies of Chen et al. (2018) and Keinan et al. (2009).

### 2.6 Demography

To calculate the change in the effective population size, we used fastsimcoal2 ver. 2.6 (Excoffier et al. 2021) and we generated the site frequency spectrum from Dataset 2 using easySFS (https://github.com/isaacovercast/easySFS) setting a projection value of 86, based on the maximum number of segregating sites recommended in the manuals of easySFS's manual (https://github.com/isaacovercast/easySFS) and Gutenkunst et al. (2009). Each of the five population demographic models (Figure S2) was simulated with the options (‐n 100,000 ‐M ‐m ‐q ‐L40) for 100 replications. The best model and parameters with the highest estimated log‐likelihood value were chosen, then 100 bootstrap iterations were run with the same options.

### 2.7 Correlation Between Genetic Distance and Elevation

Before calculation, we downloaded the elevation data in Hokkaido from elevation and slope 1‐km mesh data published by the Ministry of Land, Infrastructure, Transport and Tourism of Japan (https://nlftp.mlit.go.jp/ksj/gml/datalist/KsjTmplt‐G04‐a.html).

The Mantel test (Mantel 1967) and partial Mantel test (Smouse et al. 1986) were performed for evaluation based on correlation between genetic distance and difference in elevation. We determined the following parameters based on four hypotheses: (a) Euclidean distance, (b) high elevation, (c) low elevation, and (d) high elevation difference act as barriers to bear dispersal. We calculated five values for all pairs of sampling locations under each hypothesis: (a) Euclidean distance between two individuals, (b) the mean elevation and the highest elevation on the line segment connecting two individuals, (c) the difference between the lowest elevation on the line segment connecting two individuals, and (d) the difference between the highest and lowest elevation on the line segment connecting two individuals and its standard deviation (SD). This concept and methodology were based on that of Ohnishi et al. (2019). In addition, we calculated pairwise genetic distance using three datasets: mtDNA (Matsuhashi et al. 1999), Y‐chromosomal DNA (Hirata et al. 2017), and autosome (Dataset 3). We applied the Bray–Curtis percentage dissimilarity for mtDNA and Y‐chromosomal DNA data as the genetic distance between individuals using the ecodist package ver. 2.0.9 in R ver. 4.2.1 (R core team, 2022). Genetic distance for autosomes based on identity‐by‐state was calculated using the –1‐ibs option in PLINK.

Mantel and partial Mantel tests for genetic distance and candidate elevation parameters were performed with 999 permutations using the vegan package ver. 2.6.4 in R with the options method = spearman and permutations = 9999. In addition, ResistanceGA (Peterman 2018) was run to evaluate how the elevation impeded brown bear dispersal in Hokkaido. We used the GA.prep() function with the option (select.trans = “A”) to apply all eight transformations for optimizing elevation. Pairwise cost distances were then calculated based on random‐walk commute times, which are equivalent to CIRCUITSCAPE resistance distances, using the gdist.prep() function with the option (method = “commuteDistance”).

### 2.8 Correlation Between Genetic Distance and Climate Dynamics

To estimate the relative habitat suitability change along with past climate change, we constructed a species distribution model for the brown bears based on the current occurrence data and climate data (temperature and precipitation). We downloaded the occurrence data, described only as “HUMAN_OBSERVATION,” from 1979 to 2013 north of the equator from the Global Biodiversity Information Facility (GBIF, https://www.gbif.org, https://doi.org/10.15468/dl.mmst2x), then filtered the data using the spThin package ver. 0.2.0 in R with the option thin.par = 10. The current climate data published in Karger et al. (2017) was downloaded from the PaleoClim database (http://www.paleoclim.org/; Brown et al. 2018). We chose seven variables (bio7, 8, 9, 15, 17, 18, and 19) from the current available BIOCLIM variables (Booth et al. 2013), which were not correlated with other available variables (|*r*| < 0.7) based on pairwise correlations using the ENMTools package ver. 1.0.7 in R (Warren et al. 2021).

We evaluated multiple species distribution models based on the maxent.jar Java program (Phillips et al. 2006) using the ENMeval package ver. 2.0.4 in R (Muscarella et al. 2014) with the options, method = “randomkfold,” algorithm = “maxent.jar,” fc = c(“L”, “LQ”, “LQH”), and RMvalues = seq(0.5, 3, 0.5), and chose the model with the lowest AICc value. Ten thousand background points were randomly selected as absence data. The model was predicted using cloglog transformation of Present (Karger et al. 2017), mid‐Holocene (8.326–4.2 ka) (Fordham et al. 2017), Last Glacial Maximum (ca. 21 ka) (Karger et al. 2023), and Last Interglacial (ca. 130 ka) climate data (Otto‐Bliesner et al. 2006). For the mid‐Holocene and Last Glacial Maximum, three additional surfaces based on different climate models were used: the Community Climate System Model ver. 4, CCSM4 (Gent et al. 2011); the Model for Interdisciplinary Research on Climate—Earth System Model, MIROC‐ESM (K‐1 Model Developers 2004); and the Max Planck Institute Earth System Model for paleoclimate, MPI‐ESM‐P (Liepert and Lo 2013), downloaded from the WorldClim database version 1.4 (Hijmans et al. 2005).

## 3 Results

### 3.1 Genotyping

After applying site filtration to generate the optimal genotype dataset (Figure S1) for each population genetic analysis, we used four datasets: Dataset 1 (*n* = 43, 14,934,650 autosomal sites), Dataset 2 (*n* = 43, 11,136,995 autosomal sites), Dataset 3 (*n* = 43, 11,280,710 autosomal sites), and Dataset 4 (*n* = 43, 72,926 autosomal sites; *n* = 41, 673 X‐chromosomal sites). No significant bias in basic population genetic statistics, such as nucleotide diversity and depth, was found (Tables S1 and S2).

### 3.2 Genetic Structure and Gene Flow

**FIGURE 2:**
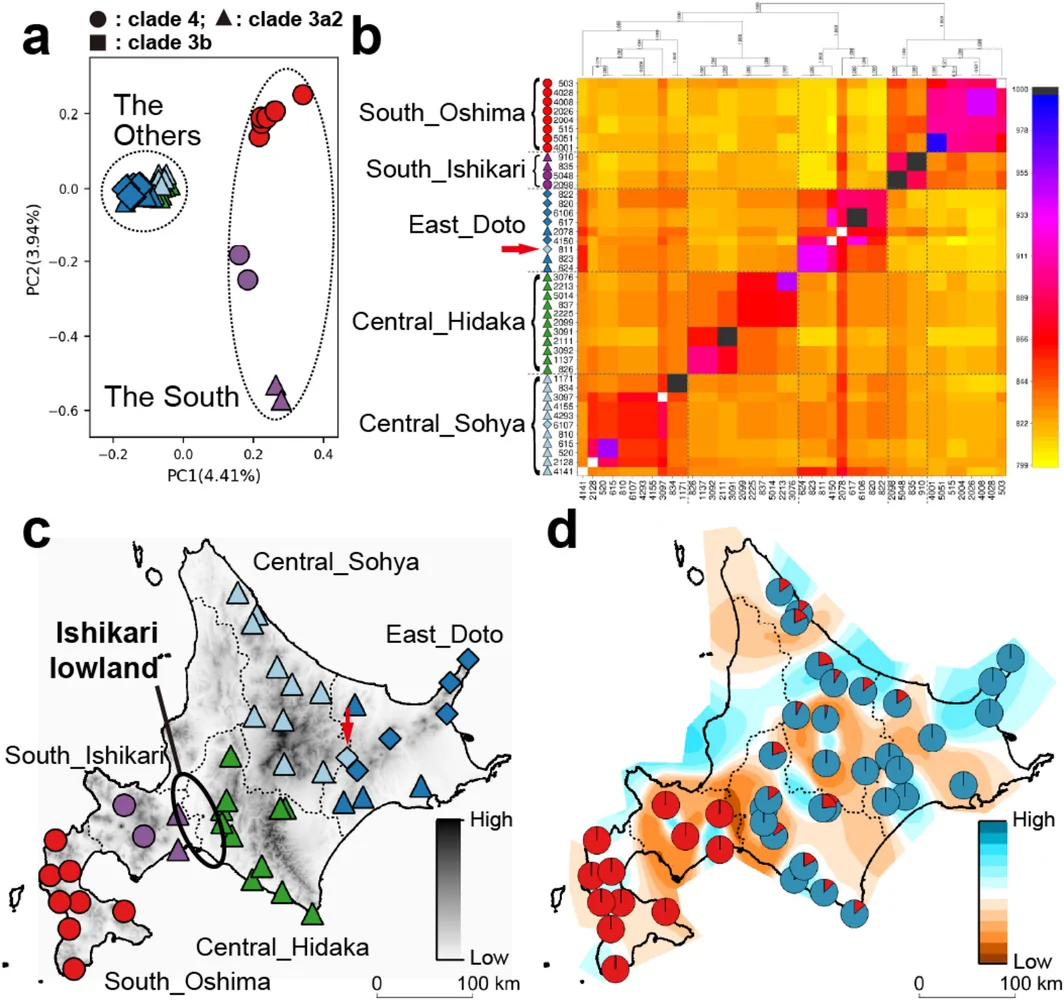
Genetic structure of the brown bear subpopulations in Hokkaido, Japan. The dot colors mean the subpopulation. Red corresponds to South_Oshima, purple to South_Ishikari, green to Central_Hidaka, light blue to Central_Sohya, and blue to East_Doto. The dot shape means the mtDNA haplotypes (Matsuhashi et al. 1999) (circle: clade 4, triangle: clade 3a2, square: clade 3b). The arrows shown in Figure 2b,c mean the individuals categorized into different subpopulations from the sampling location by fineRADstructure. The result of PCA (a), fineRADstructure (b), and ADMIXTURE (*K* = 2) and EEMS (d) are shown, respectively. The elevation in Hokkaido is shown in (c).

The results of the genetic heterogeneity analyses indicated genetic differentiation among subpopulations (Figure 2; *n* = 43, 72,926 autosomal sites). Different colors represented the different subpopulations, which corresponded to the designated management units (Oshima peninsula, South_Oshima; Shakotan‐Eniwa, South_Ishikari; Hidaka‐Yubari, Central_Hidaka; Doto‐Sohya *a*, Central_Sohya; Doto‐Sohya *b*, East_Doto; and Teshio‐Mashike, Central_Teshio). The South (South_Oshima and South_Ishikari) was clearly separated from the others (Central_Hidaka, Central_Sohya, and East_Doto) (Figure 2a), which is consistent with the boundary between mtDNA lineages clade 4 and clade 3a2. On the other hand, the boundary between mtDNA lineages clade 3a2 and clade 3b was not clearly supported by autosomal variants. The fineRADstructure results (Figure 2b) also revealed the grouping of the two subpopulations and weak genetic differences between Central_Sohya and East_Doto because of the absence of a defined separation (red arrows shown in Figure 2b–d), which was also supported by the lowest difference in *F*_st_ values between Central_Sohya and East_Doto (Table S3). While differences in *F*_st_ values (*F*_st_ = 0.078–0.089) among South_Ishikari and other subpopulations were relatively higher (Table S3), average *d*_xy_ values indicated greater differentiation between South_Oshima and the other subpopulations, excluding South_Ishikari (*d*_xy_ = 0.2556–0.2559; Table S4).

The ADMIXTURE clustering analysis (*n* = 43, 72,926 autosomal sites) revealed that a single population (*K* = 1) is the most likely based on the cross‐validation error (CV error; Table S5). At the same time, the results of *K* = 2, 3, and 4 (Figures 2d and S3) also showed the difference between the South and the other subpopulations indicated by the results of the PCA and fineRADstructure analyses. For example, *K* = 2 showed the two subpopulations, the South and the other subpopulations (Figure 2d).

Compared the results of the EEMS shown in Figure 2d (*n* = 43, 14,934,650 autosomal sites) with the elevation in Hokkaido (Figure 2c), the lower gene flow rate seemed to be more frequent in the lowlands, particularly in the Ishikari lowland, than in the highlands.

All *Q* statistics among the subpopulations in Hokkaido (*n* = 43, 72,926 autosomal sites; *n* = 41, 673 X‐chromosomal sites) were lower than 0.75. If there is no sex bias in the effective population size or dispersal, the *Q* statistic value converges at 0.75 (Chen et al. 2018; Keinan et al. 2009). Thus, the genetic differentiation of X‐chromosomal DNA was higher than the expected value under no sex‐biased in effective population size, dispersal, and mating (*Q* values = 0.324–0.463; Table S6). Within the Hokkaido population, *Q* statistics between Central_Sohya and East_Doto, whose boundary is near to the boundary between clade 3a2 and clade 3b, are lowest although the range of 95% CI overlaps despite the overlap of the 95% confidence intervals.

### 3.3 Demography

**FIGURE 3:**
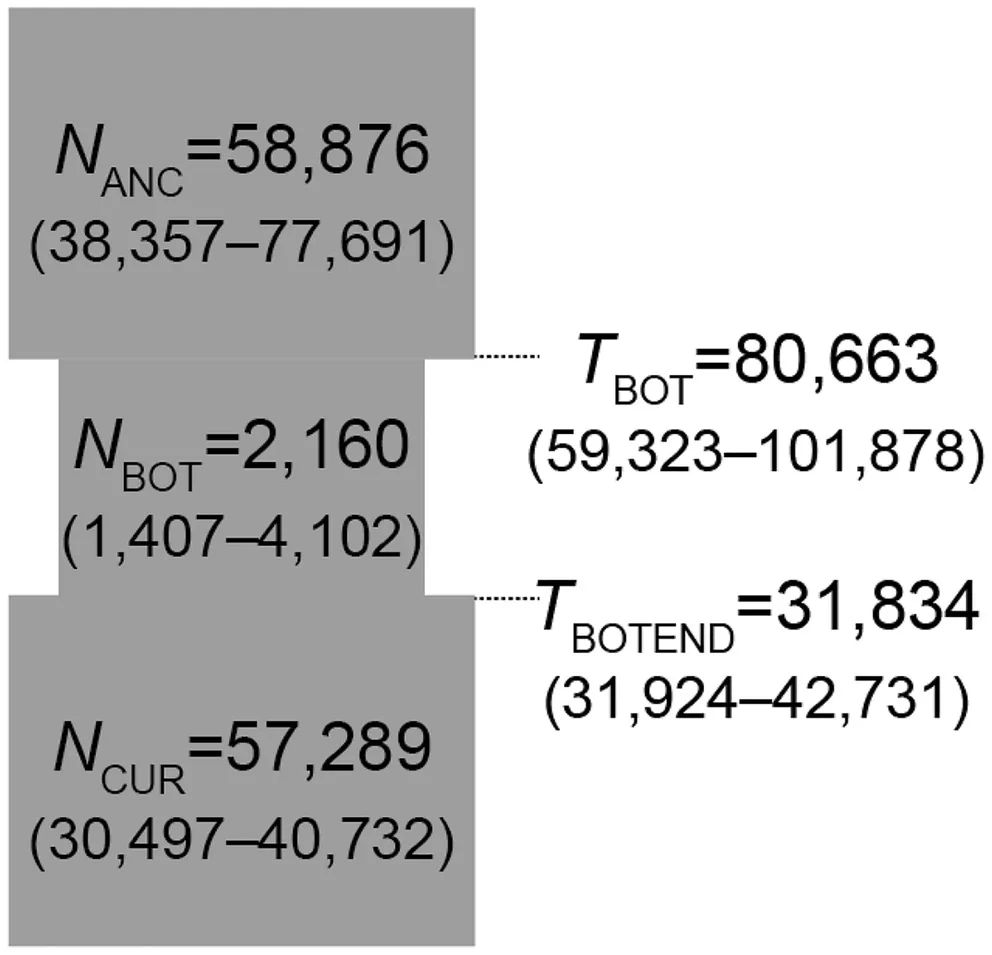
Best supported demographic model by fastsimcoal2. *N*_x_: Number of diploid individuals included in the effective population. *T*_x_: Years ago. Years were calculated under the generation time being set as 11 years (Benazzo et al. 2017; Cronin et al. 2009).

To estimate population demography after migration into Hokkaido, we simulated five models: (a) one expansion, (b) one decline, (c) two expansions, (d) two declines, and (e) one bottleneck (Figure S2) using fastsimcoal2 (*n* = 43, 11,136,995 autosomal sites). A model in which one bottleneck has happened was most statistically supported based on the maximum observed likelihood values (Table S7). When the generation time was set at 11 years (Benazzo et al. 2017; Cronin et al. 2009), the start of the decline was 80,663 years ago (95% CI: 59,323–101,878), and the end was 31,834 years ago (95% CI: 31,924–42,731) (Figure 3). These times corresponded to the Last Glacial Periods (LGP; approximately 70,000–10,000 years ago).

### 3.4 Correlation Between Genetic Distance, Landforms, and Climate Dynamics

As a result of EEMS (shown above), we assumed that the lowland acted as a barrier to dispersal for the Hokkaido population over tens of thousands of years. Two calculations, the Mantel and partial Mantel test, were done to investigate the assumption. The tests assessed the correlation between pairwise genetic distance and the elevation on the line segment connecting two individuals. Mantel and partial Mantel tests based on mtDNA revealed that, within pairs, the lower the elevation, the higher the genetic differentiation of the mtDNA, though the correlation was also influenced by isolation by distance (Tables S8 and S9). Using the autosomal dataset, both tests supported only isolation by distance. For the Y‐chromosomal data, only the Mantel test indicated a weak correlation between the genetic differentiation and the high elevation between two individual pairs. Several previous studies have suggested the limitation of Mantel and partial Mantel tests (Balkenhol et al. 2009; Meirmans 2012), so we used ResistanceGA to investigate whether the highland or lowland act as barriers to bears' migration in Hokkaido. Based on mtDNA and autosomal variants, the AICc values for models incorporating elevation were lower than those for both the null model and the model incorporating geographic distance (Table 1). Additionally, the optimized resistance values were higher in lowland areas than in highland areas (Figure S4). The results indicated that the lowland in Hokkaido acts as a barrier for mainly females (Table 1, Figure S4). Conversely, when using the Y‐chromosomal data, isolation by distance was more strongly supported as a barrier than the elevation (Table 1), which means males are less affected by altitude as a barrier than females.

**TABLE 1:** Results by resistanceGA.

Surface	obj.func_LL	k	AIC	AICc	ΔAICc	ωi	R2m	R2c	LL	Equation
Autosome										
Elev	7864.324983	4	−15720.65	−15719.5973	0	0.95145123	0.35571963	0.84200209	7864.32498	Inverse Monomolecular
Distance	7858.973248	2	−15713.9465	−15713.6465	5.95083924	0.04854877	0.26012615	0.81011648	7858.97325	
Null	7603.637612	1	−15205.2752	−15205.1777	514.419672	1.877E‐112	0	0.58678005	7603.63761	
mtDNA										
Elev	1391.352687	4	−2774.70537	−2773.79628	0	1	0.69497836	0.95896767	1391.35269	Inverse Monomolecular
Distance	1163.260267	2	−2322.52053	−2322.25966	451.536618	8.914E‐99	0.52348297	0.69325959	1163.26027	
Null	732.8371096	1	−1463.67422	−1463.58911	1310.20717	3.105E‐285	0	0.3102302	732.83711	
chrY										
Distance	7640.15639	2	−15276.313	−15276.077	0	0.90808719	0.06463797	0.41187112	7640.15639	
Elev	7640.15639	4	−15272.313	−15271.496	4.581	0.09191281	0.06463797	0.41187112	7640.15639	Distance
Null	7591.42984	1	−15180.86	−15180.783	95.294	1.842E‐21	0	0.31956679	7591.42984	

**FIGURE 4:**
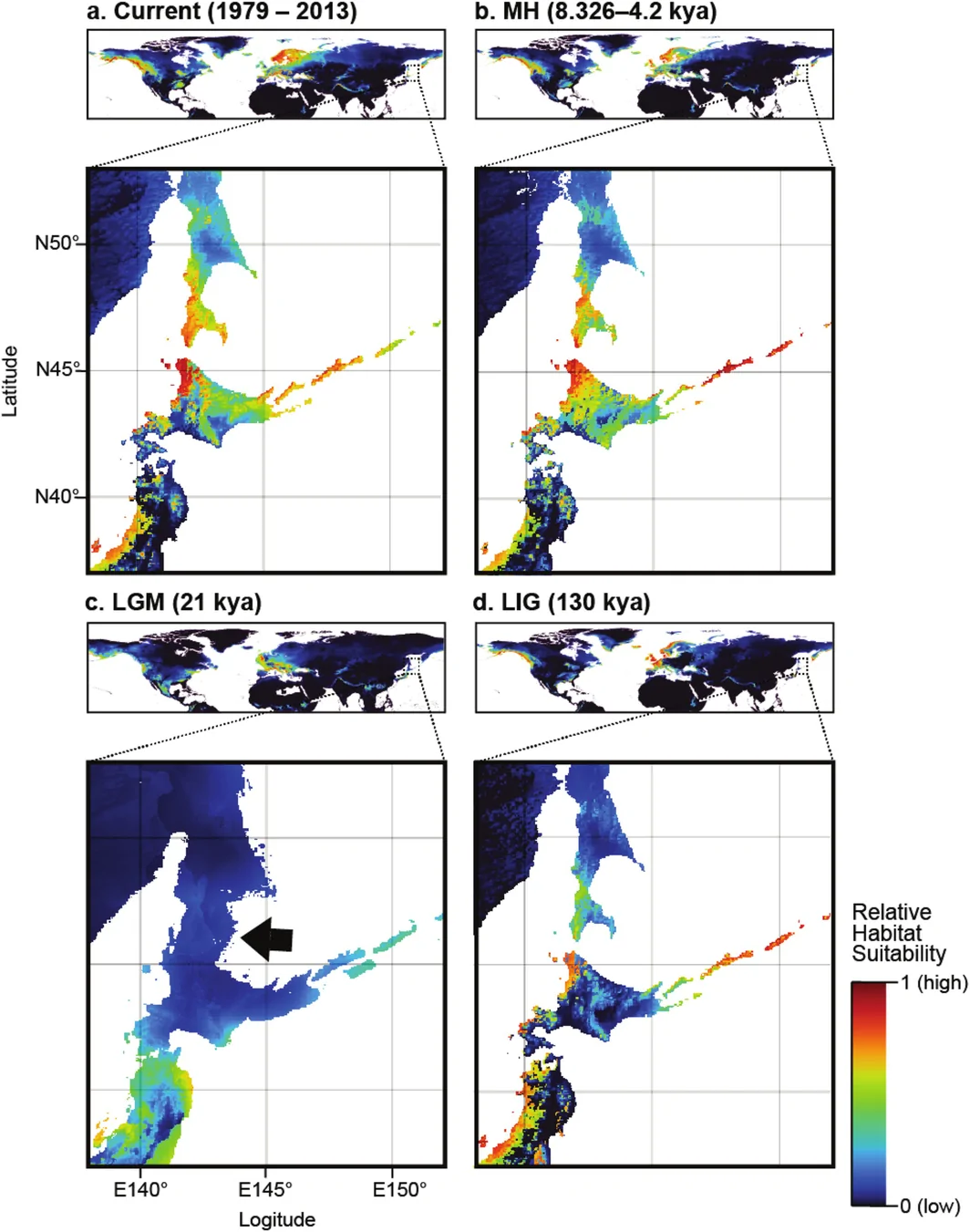
Inferred change of relative habitat suitability for brown bears using Maxent. Each picture shows the result of the Current (a), the Middle Holocene (b), the Last Glacial Maximum (c), and the Last Inter Glacial (d). The black arrow in Figure 4c means the Soya land bridge. kya: thousands of years ago.

We constructed a species distribution model to estimate the relative habitat suitability change along with past climate changes. By evaluating several species distribution models, a Maxent model with linear, quadratic, and hinge features, with RM values of 0.5, was chosen as the best model based on its AICc value (Table S10). According to the contribution rates (Table S11), the best distribution model was primarily explained by three environmental factors: BIO17 (Precipitation of the Driest Quarter), BIO8 (Mean Temperature of the Wettest Quarter), and BIO7 (Temperature Annual Range). Two of these variables are temperature‐related, and their response curves were unimodal (Figure S5), a pattern commonly observed in species' environmental response curves. The relative habitat suitability (BIO7, 8, 9, 15, 17, 18, and 19) during the Last Glacial Maximum (LGM) approximately 21,000 years ago was estimated to be low in Hokkaido (Figure 4). This inference was also supported when the different climate model surfaces were used (Figures S6 and S7).

## 4 Discussion

In this study, we aimed to investigate the colonization history of the Hokkaido population, in relation to genetic structure mismatches between mtDNA and the Y‐chromosome, then evaluate the influence of the male‐biased dispersal and environmental factors on the population demography. The results showed that the Hokkaido population is divided in two cluster, namely the South (South_Oshima and South_Ishikari) and the other subpopulations (Central_Hidaka, Central_Sohya, and East_Doto). This genetic differentiation partially corresponds to the mtDNA genetic structure (e.g., the boundary between clade 4 and clade 3a2), but the boundary between clade 3a2 and clade 3b was not clear. Although admixture between the Hokkaido subpopulations was ongoing (de Jong et al. 2023; Endo et al. 2021), a difference in gene flow between the subpopulations was supported by ADMIXTURE and EEMS, and probably influenced by the lowlands, mainly for females. In addition, the Hokkaido population was inferred to have experienced bottlenecks and a recent expansion mainly by males. In addition, relative habitat suitability for brown bears in Hokkaido was low in the LGM.

### 4.1 Population Demography Based on Autosomal and X‐Chromosomal Variants

All *Q* statistics values of ddRAD‐seq datasets were lower than the expected value, 0.75. Sex‐biased processes, which differ in effective population size between females and males, are known to cause the *Q* statistics values to deviate significantly from 0.75 (Keinan et al. 2009). Notably, a sex bias in dispersal, lifespan, and breeding opportunity influences the *Q* statistics values (Webster and Wilson Sayres 2016). In the case of the brown bear, *Q* statistics would be lower than 0.75 because of male‐biased dispersal. The sex ratio and difference in lifespan between sexes, in which the number of females is slightly larger than that of males (Sato 2015; Shimozuru et al. 2022), could also be reasons for affecting *Q* statistics values. If so, *Q* statistics could be higher in both situations because the effective female population size is larger than that of males. In addition, there is no confirmed significant bias in the sex ratio in the brown bear (Swenson et al. 1994), or difference in reproductive success between sexes (Shimozuru et al. 2020). Thus, the results of our study can be explained by male‐biased dispersal.

### 4.2 Genetic Structure of the Hokkaido Population Based on Nuclear Genomic Data

The results of PCA, ADMIXTURE (*K* = 2, 3, and 4), and fineRADstructure analysis in our study detected genetic heterogeneity in Hokkaido. Significantly, the difference between the South (South_Oshima and South_Ishikari) and the other subpopulations (Central_Hidaka, Central_Sohya, and East_Doto) was highest (*F*_st_: 0.047–0.089; *d*_xy_: 0.2540–0.2559; Figure 2a), which was also shown by a previous study (Endo et al. 2021). This result could reflect that the ancestor from the South was mainly the clade 4 of mtDNA. The isolation of the South from the other regions is more likely to have happened because of the Ishikari lowland, at the boundary of the two subpopulations, and the contraction of suitable habitat in Hokkaido in the LGM (Figure 4c). On the other hand, differentiation within the other subpopulations (Central_Hidaka, Central_Sohya, and East_Doto) was low (*F*_st_: 0.021–0.033; *d*_xy_: 0.2488–0.2519), even though the boundary between mtDNA lineages was also present. Considering the low *Q* statistics within the subpopulations (0.324–0.463), the effect of male‐biased dispersal is likely to be especially pronounced compared to the South subpopulations (South_Oshima and South_Ishikari).

Our demographic simulation using fastsimcoal2 indicated that the Hokkaido population experienced a decline in the effective population size 22,616 years ago (95% CI: 2972–71,942) and a recent rapid expansion. Hirata et al. (2013) reported that the coalescence times of three mtDNA lineages in Hokkaido were approximately several tens of thousands of years ago (27,000–42,000 years ago). In addition, the common ancestor of the Hokkaido population was estimated to occur approximately 53 thousand years ago based on Y‐chromosomal analysis (Hirata et al. 2017). The time estimate for the population decline in this study is consistent with these times. The time is also closer to the LGP, when many mammals moved on a large scale (Fu and Wen 2023). Therefore, the decline might have occurred because of climate change in the LGP, which is also supported by the lack of fossil records of brown bears on Hokkaido in the Late Pleistocene age (Takakuwa et al. 2007).

### 4.3 Correlation Between Genetic Distance, Landforms, and Climate Dynamics

Our study revealed geographical barriers to population colonization over a period of several thousand years by searching for a correlation between landforms and genetic distance based on maternal and paternal heritable markers from previous studies (Hirata et al. 2017; Matsuhashi et al. 1999) and nuclear variants. We found a negative correlation between low elevation and genetic distance for the mtDNA and autosomal variants, but no correlation for the Y‐chromosomal DNA.

We found that gene flow between the South (South_Oshima and South_Ishikari) and the other subpopulations seems to be restricted by a wide lowland; the Ishikari lowland that separates them was the area of lowest gene flow within Hokkaido Island according to the results of *F*_st_ and EEMS results. Conversely, previous studies have suggested that highland and elevation variance were also barriers to dispersal for each American black bear ( *Ursus americanus* ) in North America (Cushman et al. 2006; Short Bull et al. 2011) and the Asiatic black bear ( *Ursus thibetanus* ) in Japan (Ohnishi et al. 2019). Although the partial Mantel test based on the Y‐chromosomal DNA markers indicated a weak correlation between the highland and the genetic distance, our results indicate that lowland areas have a greater influence on gene flow in the Hokkaido population than high‐elevation areas. There was a brackish water lake in the Ishikari lowland during the mid‐Holocene (Ishii and Hori 2016; Sagayama et al. 2010), together with many oxbow lakes, and widespread peatlands (Ishii and Hori 2016). Such environments were likely unsuitable for the brown bear, whose primary food resources—such as herbaceous plants, ants, berries, and oak nuts (Ohdachi and Aoi 1987; Sato et al. 2005)—are typically found in forested areas. Thus, peatland could have been one of the dispersal barriers for the brown bear. However, we could not conclude which specific factors in the lowland are barriers in this study. Multiple factors may have acted as dispersal barriers for the Hokkaido population in the lowlands over the past ten thousand years. For example, peatlands no longer dominate the lowlands of present‐day Hokkaido, but urban and agricultural development in the region is now thought to act as a barrier to bear movement (Palm et al. 2023). To identify the barriers, genetic data from hundreds to thousands of individuals across multiple time points would be required and our dataset could not be achieved. If long‐term sampling is conducted in Hokkaido in the future, this question could potentially be resolved.

While the lowland was inferred as a barrier to dispersal, the signature was not detected from the landscape genetic analysis using the Y‐chromosomal DNA data. This is because male‐biased dispersal significantly contributed to the colonization of the Hokkaido population. The genetic structure based on autosomal variants in this study partially agrees with the genetic structure of mtDNA haplotypes, which shows genetic differentiation between the South subpopulation and others, whereas there is some genetic affinity within the Hokkaido population. Thus, the genetic structure based on autosomal variants reflects both female philopatry and male‐biased dispersal. That is, within Hokkaido, the autosomal and Y‐chromosomal admixture between different areas of distribution of mtDNA lineages (Endo et al. 2021) occurred because of the greater dispersal ability of male bears.

Relative habitat suitability for brown bears estimated by Maxent was low in the LGM. The trend, in which the habitat suitability significantly changed in the LGM, was reported widely in the northern hemispheres (de Jong et al. 2023; Fu and Wen 2023; Luna‐Aranguré et al. 2020). In Japan, the Japanese macaque ( *Macaca fuscata* ) population also followed this trend (Ito et al. 2021). The northern and central areas of Hokkaido, estimated not to be suitable for brown bears during the LGM, were covered with tundra (Dobson 1994). Igarashi (2016) estimated that Hokkaido was extensively covered by coniferous forest and grassland during LGM. A previous study (Ferguson and McLoughlin 2000) reported that the population density of the population at high‐latitude areas in North America covered with taiga (Williams et al. 2004) tends to be low. Thus, the Hokkaido population could also not have been well‐suited for such areas compared to the current predominant broad‐leaved forest vegetation in Hokkaido (Igarashi 2016). Other palynological studies (Okaura et al. 2007) indicate that oak nuts, a primary food resource for the brown bear (Ohdachi and Aoi 1987; Sato et al. 2005), were scarce during the LGM. The lack of fossil records of the brown bear on Hokkaido in the Late Pleistocene (Takakuwa et al. 2007). supports the prediction that almost all areas were not suitable for the brown bear. From the results of our demographic simulation using fastsimcoal2 and prediction of habitat suitability, the Hokkaido population would have experienced a decline during the LGM because of habitat reduction caused by climate changes.

### 4.4 Population Demographic History of the Brown Bear in Hokkaido

**FIGURE 5:**
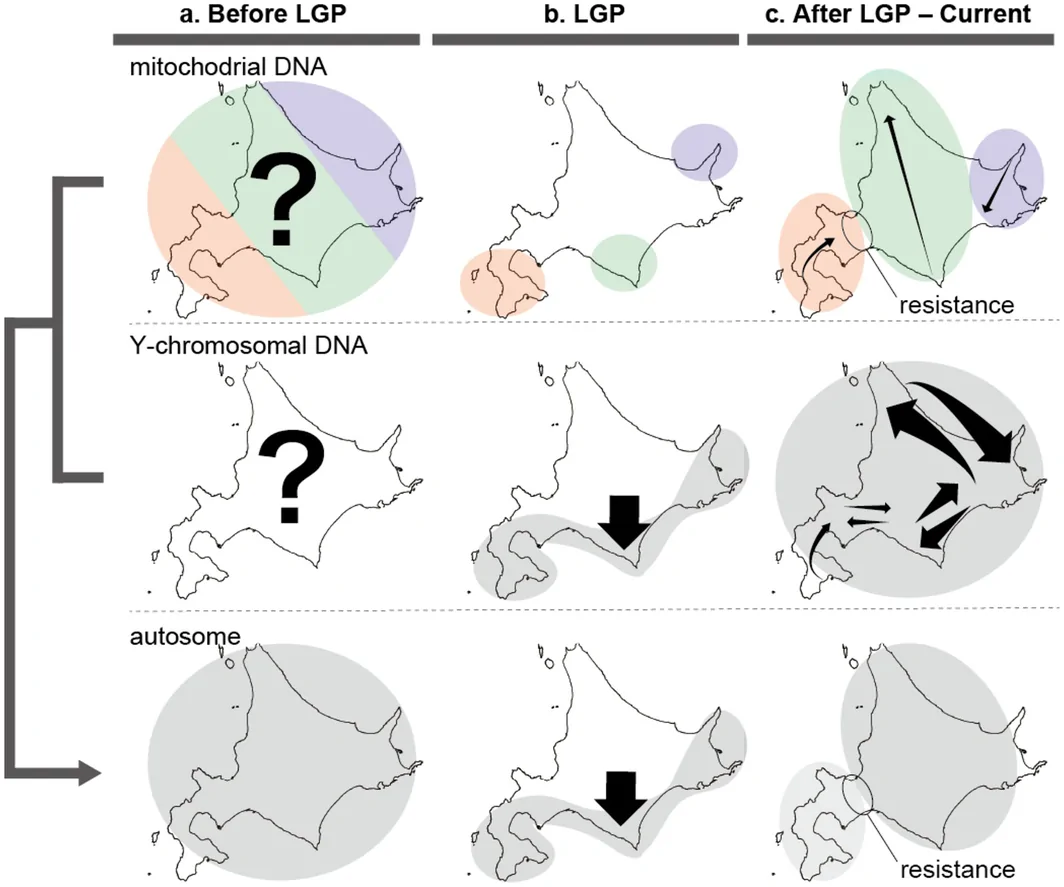
Demographic history scenario of the Hokkaido brown bear population. LGP: Last Glacial Period. Each picture means before LGP (a), LGP (b), and after LGP–Current (c).

Based on the results of this study, the following population demographic history can be inferred for the Hokkaido population (Figure 5). First, the ancestors had migrated into Hokkaido from Eurasia before the LGP. This study cannot conclude migration timing and whether different mtDNA lineages distributed separately in the past (Figure 5a). After migration, during the LGP, a population decline occurred because of a decrease in their suitable habitat, prompting the ancestors to move south and fix different mitochondrial lineages in different regions (Figure 5b). Afterward, population expansion occurred through male‐biased dispersal, resulting in distinct distributions of the three mtDNA lineages and weak spatial structure in the Y chromosome, with the nuclear genetic structure reflecting both patterns. Some restrictions on dispersal were also evident, including geographical barriers such as the Ishikari lowland (Figure 5c).

This scenario supports previous studies, suggesting (i) a lowland dispersal barrier (Matsuhashi et al. 1999), (ii) no evidence of clade 3a1, which differentiated recently within Hokkaido (Itoh et al. 2012; Matsuhashi et al. 1999; Sato et al. 2011; Shirane et al. 2018), and admixture between different lineages (Endo et al. 2021; Hirata et al. 2017; de Jong et al. 2023), and (iii) slight genetic differentiation between the South and other subpopulations in Hokkaido (Endo et al. 2021). Conversely, this scenario suggests isolation from the Eurasian continental population and a population decline despite the presence of the Soya land bridge at that time (Ohshima 1990).

Compared with the previous phylogeographic studies, our results suggest that not only sea levels but also other factors, such as climate change and landforms, are important to the biogeography of large mammals. For mammals, the population demographic history profoundly depends on the formation of land bridges resulting from sea level changes (Salis et al. 2021; Wiens et al. 2022). In particular, the species distributed in present‐day Japan could have experienced multiple land bridge formations caused by climate changes (Dobson 1994; Mckay 2012; Millien‐Parra and Jaeger 1999), thereby influencing their population demographic history. However, other environmental factors may be equally important, even for species that can easily move via land bridges, such as large mammals. We emphasize the importance of the verification of multiple factors, which would be clarified in future phylogeographic studies.

de Jong et al. (2023) proposed that the separated distributions of mtDNA lineages result from random differential fixation. Considering the results of our study, these differences could have arisen by contraction in the bears' distribution associated with climate changes after migration from the Eurasian continent to Hokkaido. To fully validate this hypothesis, the transition of haplotype frequencies should ideally be investigated via the analysis of ancient samples. Unfortunately, there is currently a lack of fossil records in Hokkaido (Takakuwa et al. 2007).

### 4.5 The Effect of Sex‐Biased Dispersal

In this study, we detected a significant contribution of male‐biased dispersal in rapid colonization, which underpins the mismatch of genetic structure patterns between mtDNA and the Y‐chromosomal DNA. Using and comparing differently inherited DNA regions made it possible to accurately detect the long‐term effect of male‐biased dispersal on gene flow over multiple generations by using tens of thousands of data points for variants with bisexual inheritance. We were able to detect subtle differentiation in male‐biased dispersal within and between populations, which was not evident when relying solely on haplotype data, such as mtDNA and the Y‐chromosomal DNA alone. Furthermore, focusing on brown bears allows for a practical evaluation of male‐biased dispersal. In the case of other animals, the influence of other factors, such as social systems, must be considered. Plant research is also challenging because most plants do not disperse long distances by themselves. Compared with other species, brown bears can be evaluated on male‐biased dispersal alone; they typically disperse over long distances and have relatively limited interactions with other individuals due to their solitary behavior. By addressing multiple challenges in evaluating sex‐biased dispersal, this study makes an important contribution to understanding the process of population formation influenced by environmental factors and the long‐term effect of sex‐biased dispersal.

### 4.6 Population Management Perspectives

The conservation and management plan of the Hokkaido population is essential. A massive decline in the number of individuals was caused by the spring cull, which was a hunting measure used in the 1990s to remove bears considered to be a potential future nuisance (Takinami et al. 2021). However, the population size has been recently stable (Japan Bear Network 2006; Hokkaido 2022), and conflict between humans and brown bears, including agricultural damage and human injuries, has become increasingly serious (Sato 2026). Thus, an effective management plan that both ensures the long‐term persistence of the Hokkaido population and mitigates human–bear conflicts is needed.

Currently, the population is managed based on the second Brown Bear Management Plan on Hokkaido (Hokkaido 2022, 2024). Setting management units based on genetic data was not suggested in this management plan, although this consideration was included in the first one. This study demonstrates that the subpopulations in Hokkaido correspond to the management units outlined in the second plan (Hokkaido 2022), with particularly clear genetic differentiation between the southern subpopulations (South_Oshima and South_Ishikari) and the other subpopulations (Central_Hidaka, Central_Sohya, and East_Doto). These results not only provide scientific support for the current management units but also suggest an additional management unit: the southern subpopulations and the other subpopulations.

We would like to suggest that it is not just how to divide units, but how to maintain connectivity between units that is important. This perspective is also supported by previous reviews on applying genetic data to wildlife management, which consider connectivity as one of the conservation principles (Andrello et al. 2022; Nielsen et al. 2022). We inferred that the lowland has been a barrier to dispersal for a long time. Currently, anthropogenic features such as buildings, roads, and farms which brown bears tend to avoid, are present in the area. Furthermore, management efforts promote spatial segregation between humans and brown bears in Hokkaido to facilitate coexistence and reduce human–bear conflicts (Sato 2026). While promoting spatial segregation between humans and brown bears, it will be crucial to maintain connectivity through lowland areas while ensuring public safety in the future management of the Hokkaido population.

## Author Contributions

**Yu Endo:** conceptualization (equal), data curation (equal), formal analysis (equal), funding acquisition (equal), investigation (equal), project administration (equal), visualization (equal), writing – original draft (equal), writing – review and editing (equal). **Naoki Osada:** supervision (equal), writing – review and editing (equal). **Tsutomu Mano:** data curation (equal), resources (equal). **Atsushi J. Nagano:** data curation (equal), writing – review and editing (equal). **Ryuichi Masuda:** funding acquisition (equal), writing – review and editing (equal).

## Funding

This work was supported by Japan Society for the Promotion of Science (Grants 18H05508, 22KJ0055) and The establishment of university fellowships towards the creation of science technology (JPMJFS2101).

## Conflicts of Interest

The authors declare no conflicts of interest.

## Supporting information


**Figure S1:** The site filtration workflow used in this study.
**Figure S2:** Five population demographic models simulated in this study using fastsimcoal2. *N*_x_: number of haploids included effective population. *T*_x_: generations ago.
**Figure S3:** Results of ADMIXTURE (right) and the distribution of the brown bears in Hokkaido, Japan (left). The shapes shown above the result of ADMIXTURE indicate the types of mtDNA lineages for each individual (Matsuhashi et al. 1999) (circle: clade 4, triangle: clade 3a2, square: clade 3b). (a) *K* = 2, (b) *K* = 3, and (c) *K* = 4.
**Figure S4:** Final optimized resistance for the mitochondria DNA (a) and autosomal variants used in this study (b). In the pictures on the left side, the color means the higher the optimized resistance value is, the darker the color, which means the darkly shaded areas indicate regions of barrier to dispersal by bears, based on each genetic distance. Dot plot in Figure S4a show mitochondrial DNA lineages (Matsuhashi et al. 1999). Pie charts in the maps show the result of ADMIXTURE (shown in Figure S3c). Pictures on the right side show the correlation between original elevation values and estimated degree of resistance.
**Figure S5:** Response curves of each environmental variance in the best model constructed by Maxent. The curves show the predicted value (*Y*‐axis) changes in each environmental variance (*X*‐axis).
**Figure S6:** Relative habitat suitability of brown bears during the Last Glacial Maximum using Maxent. Each picture shows the result using CCSM4 (a), MIROC‐ESM (b), and MPI‐ESM‐P (c). CCSM4: the Community Climate System Model ver. 4 (Gent et al. 2011). MIROC‐ESM: the Model for Interdisciplinary Research on Climate—Earth System Model (K‐1 Model Developers 2004). MPI‐ESM‐P: the Max Planck Institute Earth System Model for paleoclimate, MPI‐ESM‐P (Liepert and Lo 2013).
**Figure S7:** Relative habitat suitability of brown bears during the Middle Holocene using Maxent. Each picture shows the result using CCSM4 (a), MIROC‐ESM (b), and MPI‐ESM‐P (c). CCSM4: the Community Climate System Model ver. 4 (Gent et al. 2011). MIROC‐ESM: the Model for Interdisciplinary Research on Climate—Earth System Model (K‐1 Model Developers 2004). MPI‐ESM‐P: the Max Planck Institute Earth System Model for paleoclimate, MPI‐ESM‐P (Liepert and Lo 2013).

**Table S1:** Sampling data used in this study. Asterisks mean that each sample was included in each dataset.
**Table S2:** The result of genotyping by bcftools.
**Table S3:** Differences in *F*_st_ values among the Hokkaido population subpopulations based on autosomal data. All SE values were lower than 0.01.
**Table S4:** Differences in average *d*_xy_ values among the Hokkaido population subpopulations based on autosomal data.
**Table S5:** The values of the cross‐validation error (CV error).
**Table S6:** *Q* statistics between the subpopulations in Hokkaido. The values in the parentheses mean 95% CI calculated based on *F*_st_ and that of SE.
**Table S7:** The result of the parameters and likelihood in all models on fastsimcoal2. We converted the generation times to years and the number of genes to the number of biallelic individuals.
**Table S8:** Mantel *r* values on the Mantel test. One asterisk means the *p*‐value was lower than 0.05, and double asterisks mean the *p*‐value was lower than 0.01.
**Table S9:** Mantel *r* value on partial Mantel test. One asterisk means the value was lower than 0.05, and double asterisks mean the *p*‐value was lower than 0.01.
**Table S10:** All results to evaluate the relative habitat suitable models. The rows highlighted in gray indicate the model chosen as the best model based on AICc.
**Table S11:** Contributions of each environment variance in the best model.

## Data Availability

Raw sequence reads are deposited in the SRA (BioProject: PRJDB17752).
