# Supplementary material for: Male‐Biased Dispersal Leads to Expansion After Decline: The Hokkaido Brown Bear ( Ursus arctos ) Population Rebounds Post‐Last Glacial Period
Source: Ecol Evol. 2026 Sep 20;16(9):e74312. doi: 10.1002/ece3.74312 (PMC13590281; doi:10.1002/ece3.74312)
Supplement: Supplementary file 1 [file ECE3-16-e74312-s001.pdf]

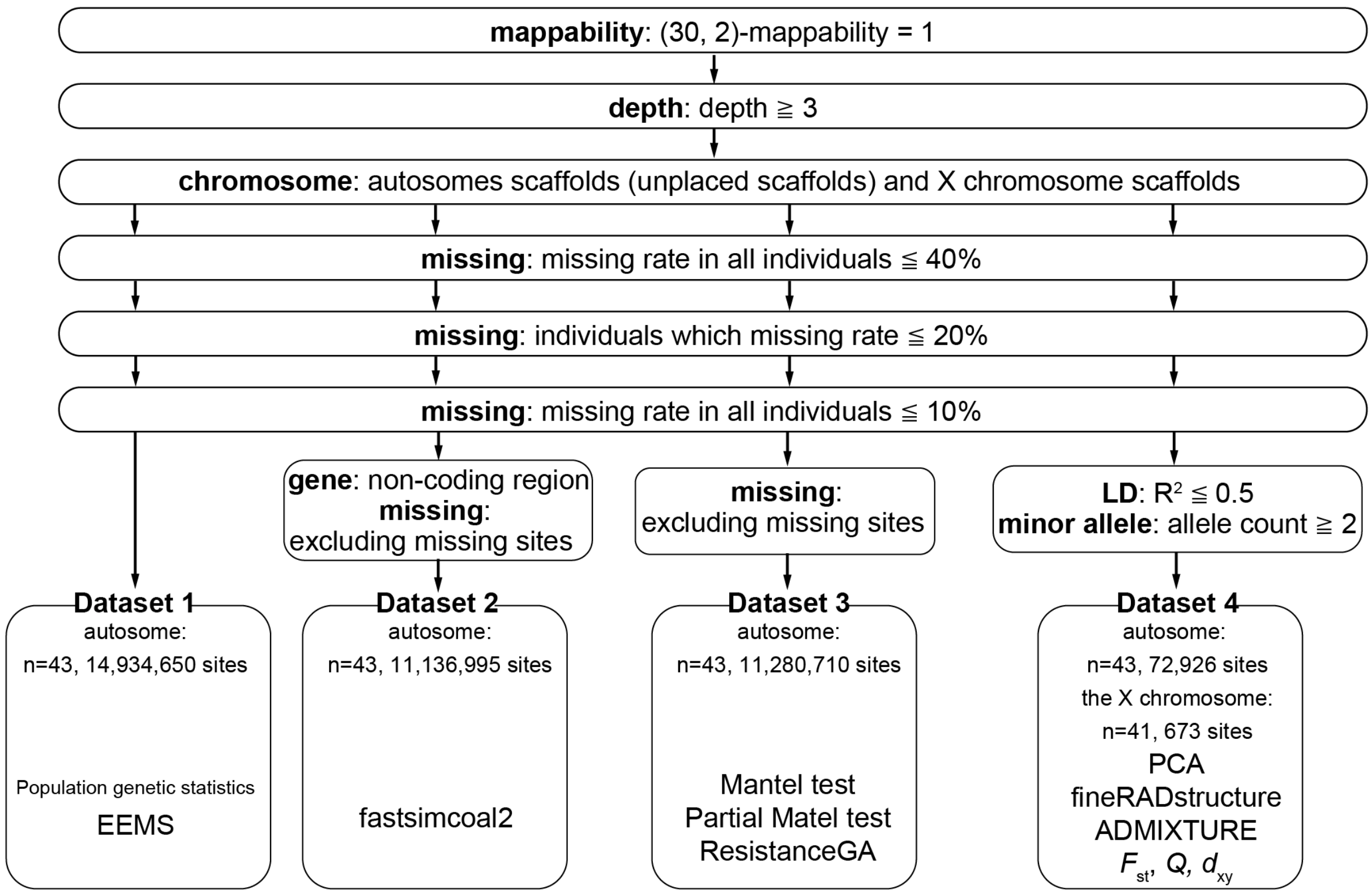

Figure S1. The single-nucleotide polymorphism (SNP) filtration workflow used in this study.

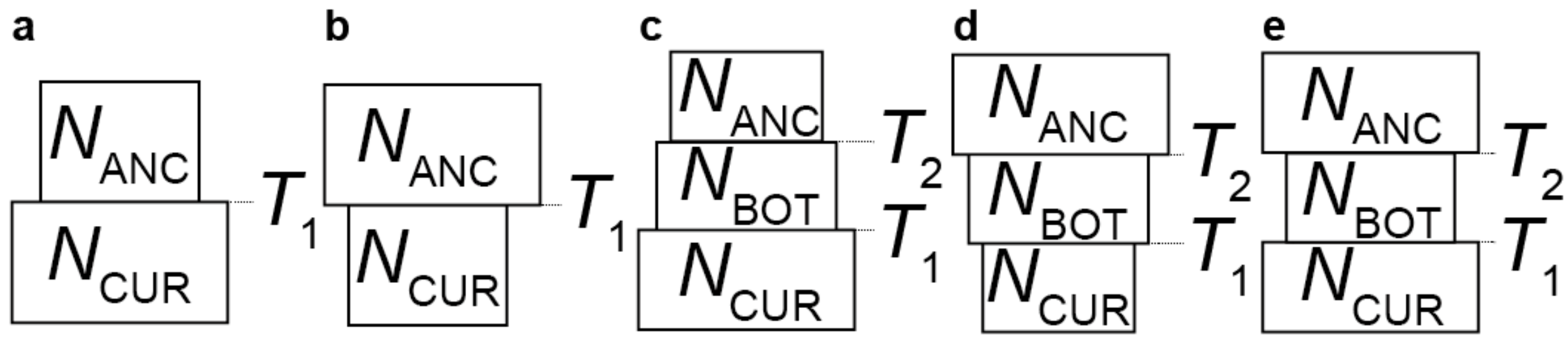

Figure S2. Five population demographic models simulated in this study using fastsimcoal2. N_x: number of haploids included effective population. T_x: generations ago.

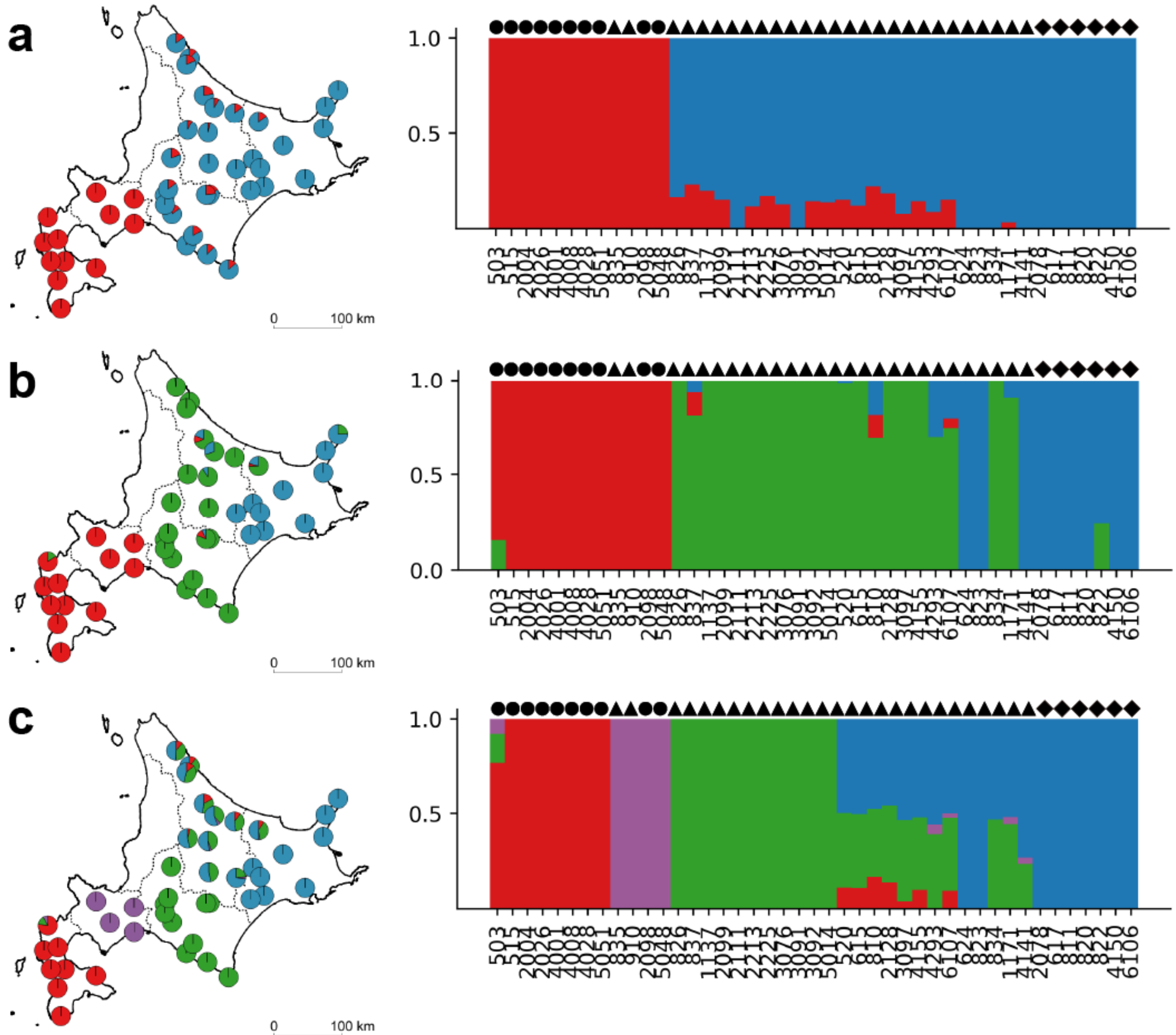

Figure S3. Results of ADMIXTURE (right) and the distribution of the brown bears in Hokkaido, Japan (left). The shapes shown above the result of ADMIXTURE indicate the types of mtDNA lineages for each individual (Matsuhashi et al. 1999) (circle: clade 4, triangle: clade 3a2, square: clade 3b). (a) K=2, (b)K=3, and (c) K=4.

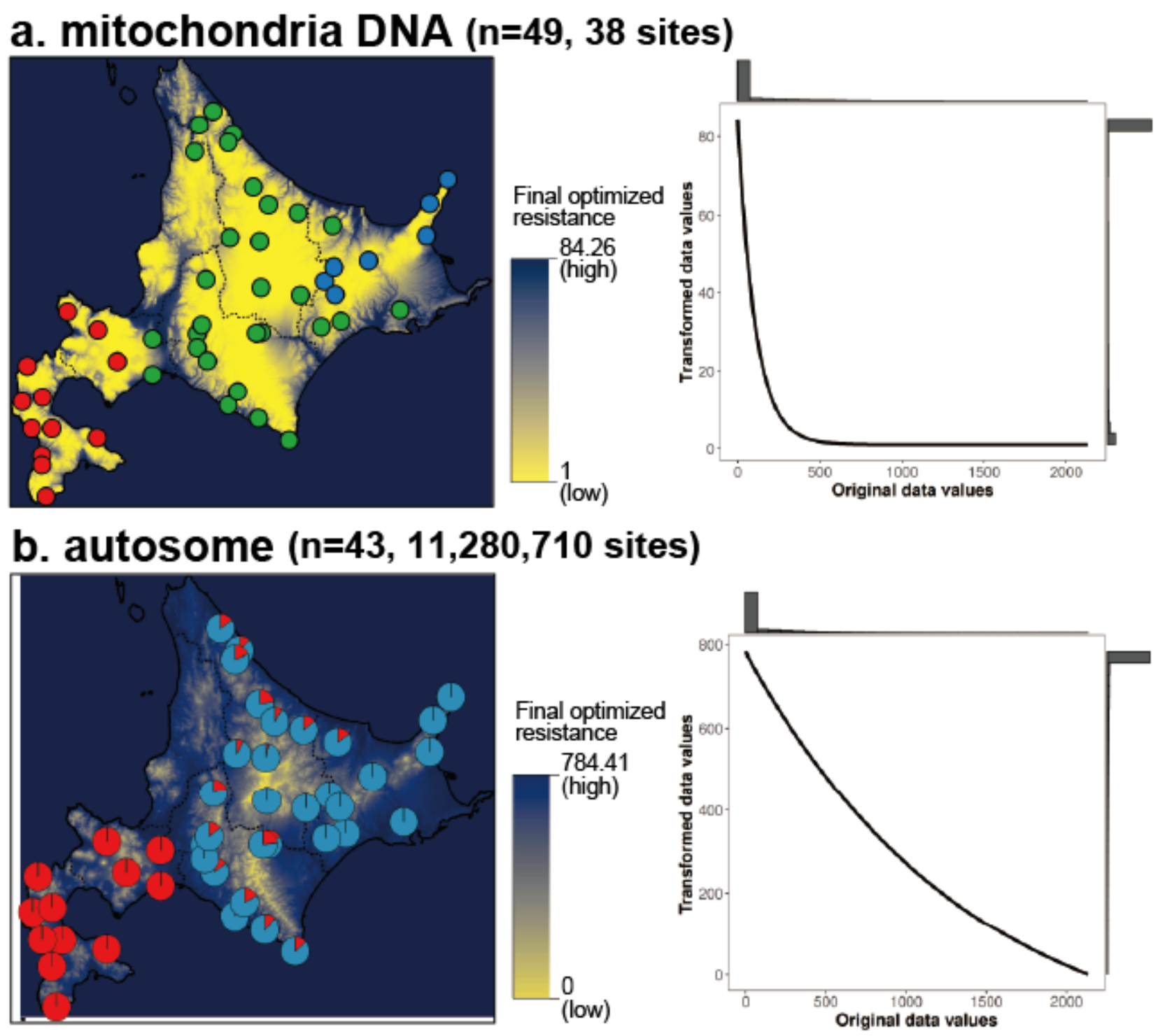

Figure S4. Final optimized resistance for the mitochondria DNA (a) and autosomal single-nucleotide polymorphism (SNP) used in this study (b). In the pictures on the left side, the color means the higher the optimized resistance value is, the darker the color, which means the darkly shaded areas indicate regions of barrier to dispersal by bears, based on each genetic distance. Dot plot in Figure S4a show mitochondrial DNA lineages (Matsuhashi et al. 1999). Pie charts in Figure S4b show the result of ADMIXTURE (shown in Figure S3c). Pictures on the right side show the correlation between original elevation values and estimated degree of resistance.

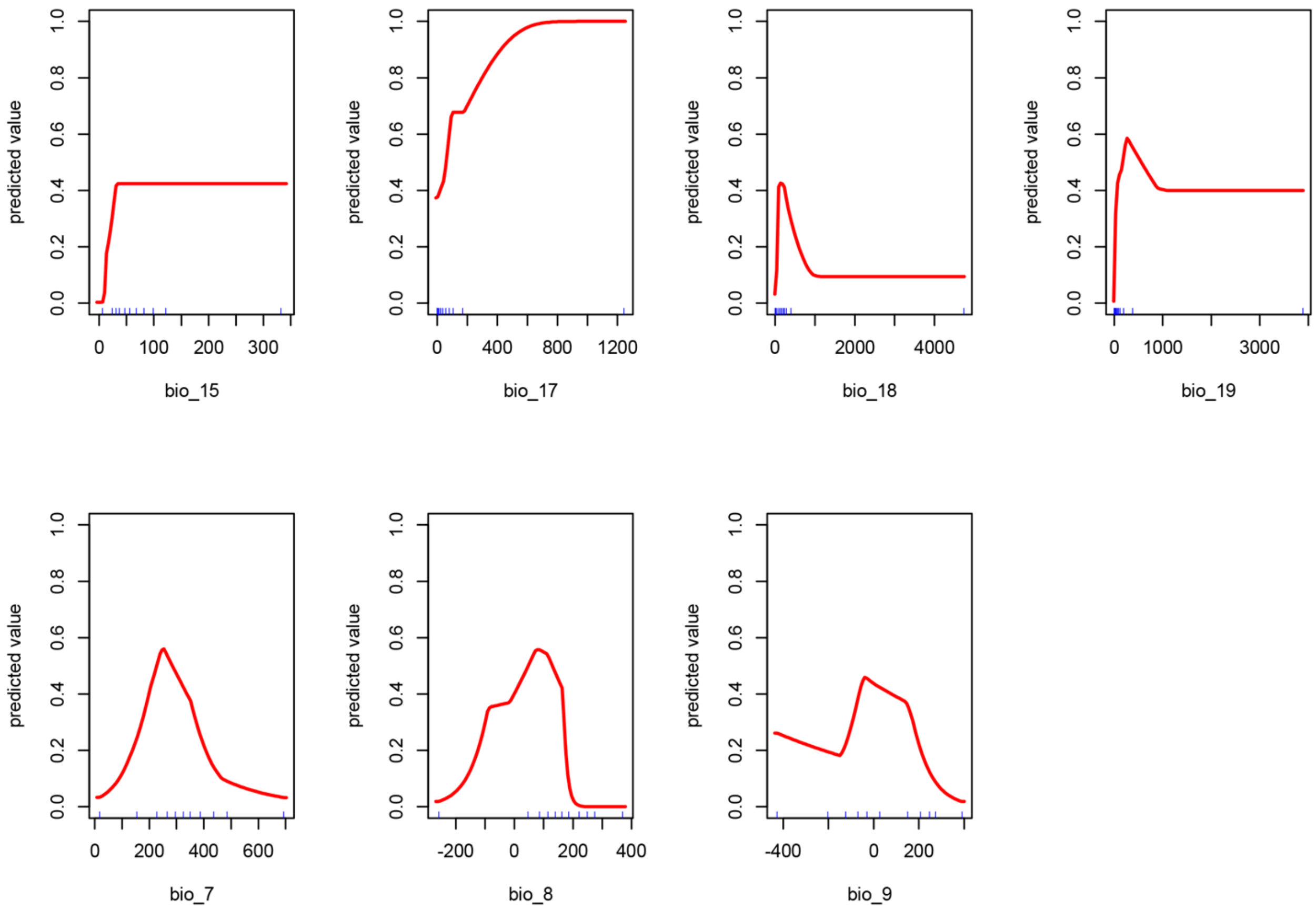

Figure S5. Response curves of each environmental variance in the best model constructed by Maxent. The curves show the predicted value (Y-axis) changes in each environmental variance (X-axis).

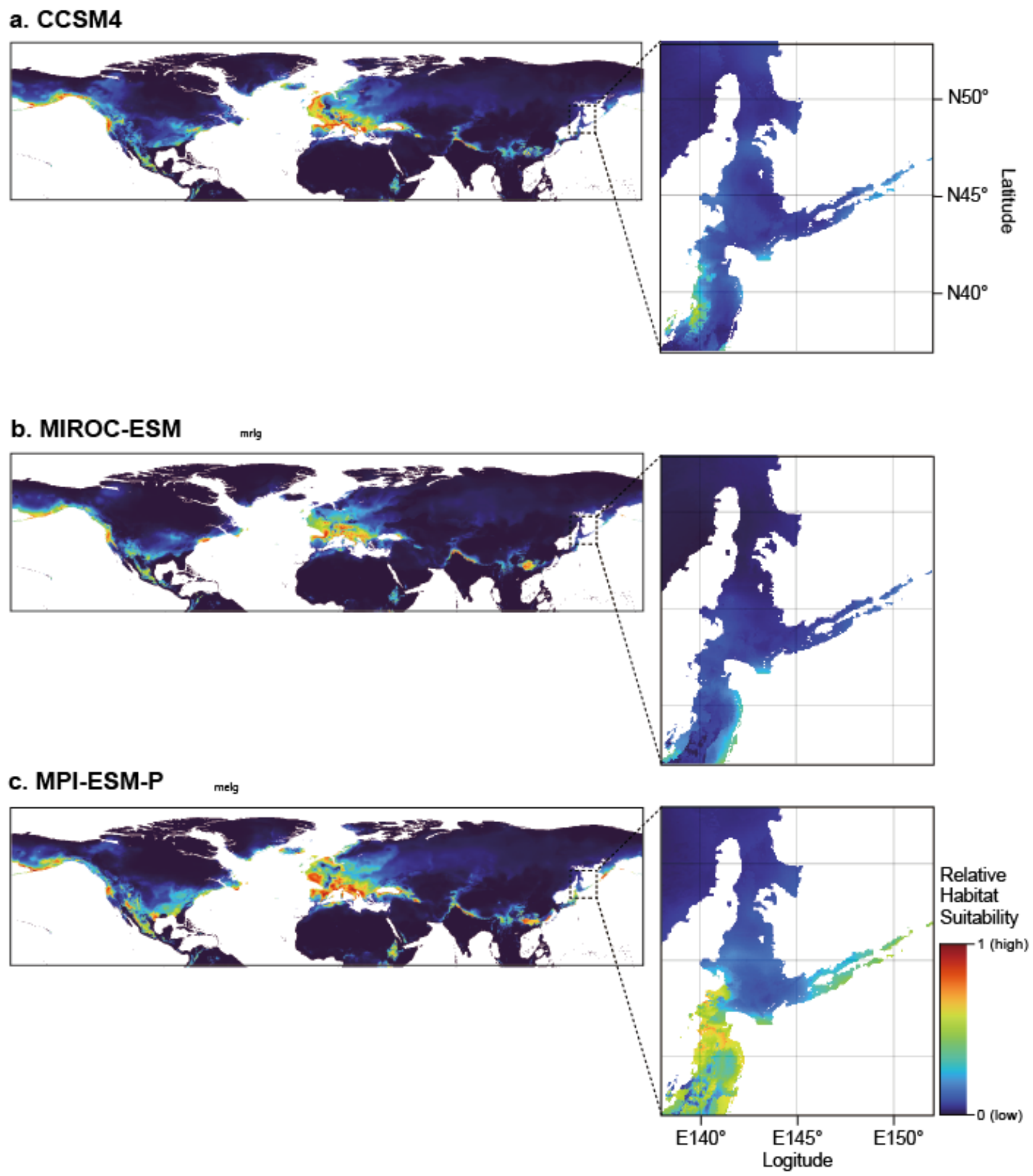

Figure S6. Relative habitat suitability of brown bears during the Last Glacial Maximum using Maxent. Each picture shows the result using CCSM4 (a), MIROC-ESM (b), and MPI-ESM-P (c). CCSM4: the Community Climate System Model ver. 4 (Gent et al. 2011). MIROC-ESM: the Model for Interdisciplinary Research on Climate – Earth System Model (K-1 model developers, 2004). MPI-ESM-P: the Max Planck Institute Earth System Model for paleoclimate, MPI-ESM-P (Liepert & Lo, 2013).

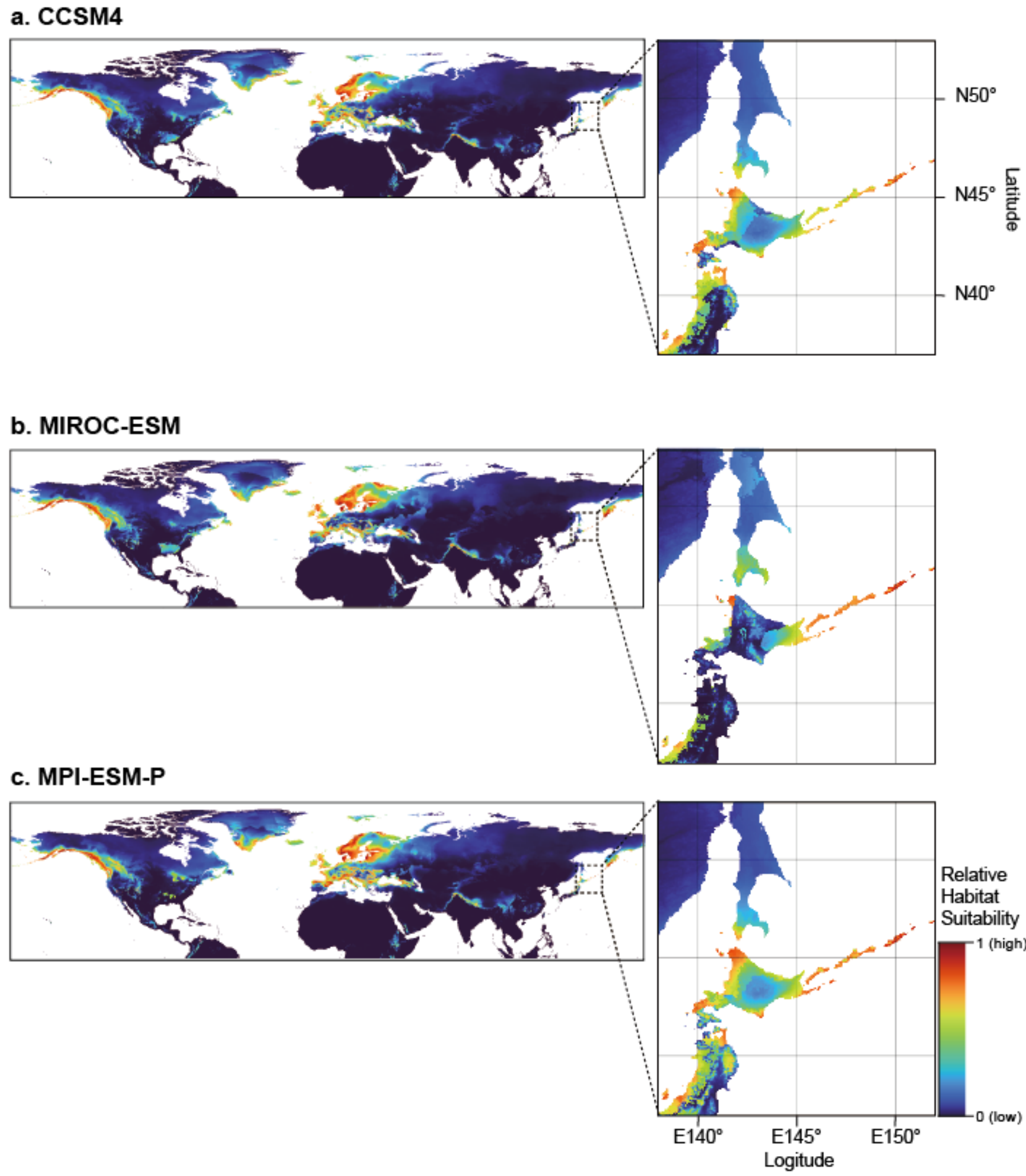

Figure S7. Relative habitat suitability of brown bears during the Middle Holocene using Maxent. Each picture shows the result using CCSM4 (a), MIROC-ESM (b), and MPI-ESM-P (c). CCSM4: the Community Climate System Model ver. 4 (Gent et al. 2011). MIROC-ESM: the Model for Interdisciplinary Research on Climate – Earth System Model (K-1 model developers, 2004). MPI-ESM-P: the Max Planck Institute Earth System Model for paleoclimate, MPI-ESM-P (Liepert & Lo, 2013).
